# Hyperspectral image analysis for CARS, SRS, and Raman data

**DOI:** 10.1002/jrs.4729

**Published:** 2015-06-14

**Authors:** Francesco Masia, Arnica Karuna, Paola Borri, Wolfgang Langbein

**Affiliations:** ^1^ School of Physics and AstronomyCardiff UniversityThe ParadeCardiffCF24 3AAUK; ^2^ School of BiosciencesCardiff UniversityMuseum AvenueCardiffCF10 3AXUK

**Keywords:** coherent Raman micro‐spectroscopy, hyperspectral image analysis, sparse sampling

## Abstract

In this work, we have significantly enhanced the capabilities of the hyperspectral image analysis (HIA) first developed by Masia *et al*. 1 The HIA introduced a method to factorize the hyperspectral data into the product of component concentrations and spectra for quantitative analysis of the chemical composition of the sample. The enhancements shown here comprise (1) a spatial weighting to reduce the spatial variation of the spectral error, which improves the retrieval of the chemical components with significant local but small global concentrations; (2) a new selection criterion for the spectra used when applying sparse sampling^2^ to speed up sequential hyperspectral imaging; and (3) a filter for outliers in the data using singular value decomposition, suited e.g. to suppress motion artifacts. We demonstrate the enhancements on coherent anti‐Stokes Raman scattering, stimulated Raman scattering, and spontaneous Raman data. We provide the HIA software as executable for public use. © 2015 The Authors. Journal of Raman Spectroscopy published by John Wiley & Sons, Ltd.

## Introduction

Raman microscopy is a powerful technique in cell imaging providing chemical specificity without the need for exogenous labels, which can alter the normal behavior of biological specimens. In the last decade, coherent Raman techniques have been developed complementing spontaneous Raman with the benefit of faster acquisition speed compatible with life cell imaging. In coherent Raman scattering, the molecular vibrations are coherently driven by the interference between two optical fields usually called pump and Stokes. In coherent anti‐Stokes Raman scattering (CARS), the pump field scattered to higher frequencies is measured free from excitation laser background, while loss (gain) of the pump (Stokes) field is detected in stimulated Raman scattering (SRS). In both techniques, hyperspectral imaging (a sequence of images of the same sample region over a range of vibrational frequencies) offers superior chemical specificity compared with the corresponding single frequency approach. In CARS, typically, the intensity is measured, and to retrieve the complex CARS susceptibility, which is the quantity linear in the chemical composition, its phase has to be determined, which requires to measure the CARS spectrum over a sufficiently large frequency range. In SRS, the imaginary part of the susceptibility is measured, so that no phase retrieval is required. However, the simple calibration of the signal amplitude relative to the non‐resonant CARS signal of glass^1^ is not available in SRS.

Once the quantity linear in the concentration is obtained, the resulting hyperspectral images need to be analyzed to achieve a quantitative chemical measurement. Different approaches have been developed in the last years to provide an efficient image visualization and to represent the coherent Raman susceptibility as a spatially resolved map of spectral components. Methods proposed in the literature use principal component analysis,^3^ hierarchical cluster analysis,^4^ independent component analysis,^5^ classical least squares analysis,^6^ and multivariate curve resolution analysis.^7^ Among the mentioned methods, multivariate curve resolution can provide a quantitative determination of the absolute concentration of the chemical substances but often needs an initial guess of the spectra.^8^ Recently, also phasor analysis^9^ has been introduced to analyze hyperspectral SRS cell images^10^ by distinguishing the different subcellular components on the basis of closeness of the corresponding phasors. The phasor approach has a low computational complexity – it uses the phases of the discrete Fourier transform of the data. It is not reducing the number of components, and it is assumed that most of the information is contained in the low frequency components, and typically, only the phase of the lowest non‐zero frequency component is analyzed, using a supervised method.^10^

We have recently developed an algorithm^1^ to analyze CARS hyperspectral images, which determine the spectra and the absolute concentration images of chemical components without prior knowledge of the spectra. The method is based on three steps. First, a singular value decomposition (SVD) is used to noise filter the data, then a phase‐corrected Kramers–Kronig algorithm retrieves the complex susceptibility from the CARS intensity, and finally, a factorization into spectra and concentrations of chemical components (FSC^3^) is performed. The first two steps are specific to the CARS data and are used to retrieve the complex susceptibility, which is linear in the chemical composition.

The FSC^3^ method is based on a non‐negative matrix factorization (NMF) algorithm,^11^ which minimizes the error in the factorization using only non‐negative matrix elements. The non‐negativity is a constrain which is reflecting a prior knowledge about the spectra. For the CARS susceptibility, we use its imaginary part and the spectrally averaged real part. The concentration matrix is also non‐negative, as it represents physical concentrations of components. No spatial constrain is used. The physical constrains governing the NMF allow an unsupervised retrieval of component concentrations and spectra. We additionally determine the absolute values of the spectra and concentrations by minimizing the deviation of the sum concentration at each point from unity, i.e. a filled volume, over the image.

The method implements these basic physical constrains, and the minimization is performed on the total deviation over the image. A limitation of using the total deviation is that components which are present only in a fraction of the image have a small weight in the global error, so that the corresponding local deviations are dominated by the statistical and systematic error over the rest of the image. Furthermore, in the version described in Ref. 1, the number of chemical components had to be chosen manually.

In this paper, we report on an extension of the FSC^3^ method, which addresses these limitations. Additional enhancements are introduced to improve the reproducibility and reduce the influence of systematic errors in the measured data. We also add the sparse sampling spectral reconstruction method,^2^ which we have recently demonstrated to improve the acquisition speed, which we enhanced here by a refined method to determine the number of spectra used for reconstruction, and a filter for outliers in the data using SVD, suited e.g. to suppress motion artifacts. Importantly, we provide the executable of the hyperspectral image analysis software used in this work in the supplement, for public use.

## Experiment

Coherent anti‐Stokes Raman scattering hyperspectral images have been acquired on a home‐built multi‐modal laser‐scanning microscope based on an inverted Nikon Ti‐U. A detailed description of the setup can be found in Ref. 12 Briefly, the pump and Stokes beams for CARS excitation are obtained by splitting a broadband (660–970 nm) laser beam from a 5 fs Ti:Sa laser into the wavelength ranges of 660–730 nm and 730–900 nm, respectively. The CARS signal is emitted at a frequency of 2*ω*_p_ − *ω*_S_, where *ω*_p_(*ω*_S_) is the frequency of the pump (Stokes) beam. Hyperspectral imaging is achieved by spectral focussing.^13^, ^14^, ^15^ The pump and Stokes pulses are equally linearly chirped, resulting in a constant instantaneous frequency difference within the pulse duration. The instantaneous frequency difference can be modified by controlling the delay between pump and Stokes. The vibration energies, which can be addressed in our setup, are in the range of 1200–3800 cm^−1^, and the spectral resolution is 10 cm^−1^. The data discussed in this paper were taken over a 2600–3700 cm^−1^ range with either a 20× 0.75 NA dry objective and a 0.72 NA dry condenser or a 60× 1.27 NA water immersion objective (Nikon CFI Plan Apochromat IR *λ*S series) and a 1.4 NA oil immersion condenser. The signal collection is in forward direction. The measured spatial resolutions for the CARS intensity (full width at half maximum) are 0.6 (1.1) µm and 0.25 (0.65) µm in the lateral (axial) direction, respectively. The measured spatial resolutions for the retrieved CARS susceptibility (full width at half maximum of the point‐spread function amplitude) are 0.9 (3.5) µm and 0.6 (2) µm in the lateral (axial) direction, respectively. The CARS signal is discriminated by a pair of band‐pass filters (Semrock FF01‐562/40) and detected by a photomultiplier (Hamamatsu H7422‐40). The pixel dwell time was 10 µs.

## Result and discussion

### Unsupervised weighted FSC^3^

Here, we describe an enhancement of the FSC^3^ method reported in Ref. 1 in order to improve the retrieval of chemical components present only in small fraction of the total image points, which are contributing with a low‐average concentration. We note that the FSC^3^ algorithm factorizes the hyperspectral data **D** into non‐negative concentrations **C** and spectra **S**, i.e. **D** = **C** ⊗ **S** ^T^ + **E**, minimizing the norm of the error matrix **E**. The non‐negativity of the concentrations is a physical constrain by definition. Concerning the spectra, we use the imaginary part of the CARS susceptibility, which is non‐negative in thermal equilibrium, and the non‐resonant real part, which is positive for the typical non‐resonant excitation conditions for which the electronic transitions are above the two‐photon energy of the excitation pulses. The systematic errors and random noise such as shot noise in the data lead to a lower bound of the error for each spatial point. Chemical components, which have a small average concentration, have a small influence on the error. However, if these components are concentrated into few spatial points, these points will show a large spectral error, beyond the general systematic and statistical error. To reconstruct such components localized in small regions of high concentration, we have developed an iterative scheme to favor a homogeneous spectral error instead of minimizing the global error. This algorithm introduces a weight *w*_*p*_ of the spectrum at each point *p* to adjust its importance in the NMF minimization. This weight, initially chosen to be unity for all points, is iteratively adjusted according to the spectral error $E_{Sp}=\sqrt{P\sum_{j=1}^{S}\;E_{jp}^{2}}∥D{∥}_{F}$ and the concentration error *E*_C*p*_ = (∑_*k*_ *C*_*pk*_) − 1 of the point *p* defining the weight $w_{p}^{\left(i+1\right)}$ at iteration step *i* + 1 as

(1) $$w_{p}^{\left(i+1\right)}=e^{-\beta |E_{Cp}|}{\left(w_{p}^{\left(i\right)}\frac{E_{Sp}}{{\bar{E}}_{S}}\right)}^{1-\alpha }\text{,}$$

where ${\bar{E}}_{S}$ is the average spectral error over all points, and 0 ≤ *α* < 1 and 0 ≤ *β* ≤ 1 are parameters controlling the relevance of concentration error and spectral error in the change of the weight between iteration steps. The iteration is stopped when the norm of the variation of normalized weights $\left||\frac{w_{i+1}}{\bar{w_{i+1}}}-\frac{w_{i}}{\bar{w_{i}}}|\right|$ is not reduced below any of the values of the last *j* iterations, with *j* chosen to be 3 for the data presented in this work. This iteration can lead to a divergence of the weight at specific spatial positions, for example, if they contain movement artifacts. This is accounted for by a weight threshold, which excludes spatial points from the NMF if their weight exceeds $\gamma \sqrt[{4}]{P}$, with a parameter *γ* > 0. A single point of weight $\sqrt[{4}]{P}$ has the same contribution in ||**E**|| as a number of $\sqrt{P}$ unweighted points.

In order to improve the reproducibility of the NMF, which starts from random spectra and concentrations, one FSC^3^ step in our enhanced algorithm consists of *n* independent NMFs using random initial spectra and concentrations, with a high tolerance target *τ*_H_ to provide a fast execution. The tolerance target in the NMF algorithm^11^ is the root mean square (RMS) change of **C** and **S** per iteration relative to the first iteration, and is used as stop criterion. We typically choose *n* = 10, and the NMF run which results in the smallest error is then continued with a low tolerance target *τ*_L_, resulting in an overall tolerance target of *τ*_L_ *τ*_H_ from initial random guess to result. Quantitative results on the reproducibility are discussed in the [Link]Supporting Information. The reproducibility was found to be even better using a ‘knock‐out’ method as described in the [Link]Supporting Information.

**Figure 1:**
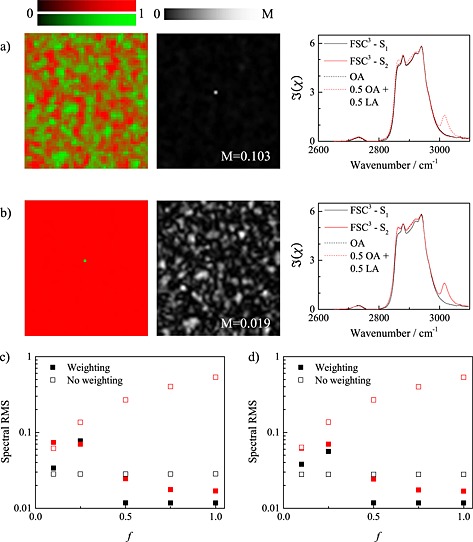
Comparison of the performance limits of the unweighted and weighted FSC^3^ methods in case of a CARS hyperspectral image made with two lipids. (a–b) Concentration maps (left) of the two FSC^3^ components mapped into the red and green values, spectral error (middle), and spectra (right) of the corresponding components (solid lines) and pure substances (dashed lines). Black (red) lines are the first (second) component and OA (LA), respectively. (a) standard FSC^3^, (b) weighted FSC^3^. *M* indicates the maximum value of the greyscale. (c–d) RMS deviation of the FSC^3^ spectra from the pure substance spectra for the unweighted (empty symbols) and weighted FSC^3^ (full symbols) as a function of the LA fraction *f* in the modified point. Black (red) symbols refer to the first (second) FSC^3^ spectrum. (c) *τ*_L_ = 10^− 3^, (d) *τ*_L_ = 10^− 5^.

The results of the weighted FSC^3^ method are compared in Fig. 1 with the non‐weighted FSC^3^. The analysis is made on CARS hyperspectral images of two homogeneous samples of octanoic acid (OA) and linoleic acid (LA) in the 2600–3700 cm^−1^ spectral range and 55 × 50 spatial points, using an SVD filter on the square root of the CARS intensity considering five important components, and retrieving the complex susceptibility using the phase‐corrected Kramers–Kronig algorithm with a time domain filter of 3 ps for the phase and 0.3 ps for the phase offset.^1^ In the resulting hyperspectral image of the imaginary part of the CARS susceptibility for the OA sample, one point has been modified to a spectral mixture with a fraction *1‐f* of the average spectrum of OA and a fraction *f* of the average spectrum of LA. For the FSC^3^ analysis, we used the spectral range of 2650–3100 cm^−1^, *k* = 2 components, *n* = 20, *τ*_H_ = 10^− 1^, and *τ*_L_ = 10^− 3^. Using *f* = 0.5, the unweighted FSC^3^ does not retrieve the modified point, as shown in Fig. 1(a) – the two resulting components have a fluctuating and spatially distributed concentration, their spectra *S*_1,2_ are both similar to the pure OA spectrum, and the spectral error shows the modified point. The weighted FSC^3^ instead recovers the spectrum of the modified point as shown in Fig. 1(b), and the spectral error is having a spatially random distribution. To investigate the retrieval *versus* spectral difference, we determined the RMS deviation between the OA and OA/LA mixture spectra and the corresponding FSC^3^ spectra for different fractions *f*. We find that the unweighted FSC does not recover the mixture spectrum also for *f* = 1 [Fig. 1(c)], even when decreasing *τ*_L_ to 10^− 5^ [Fig. 1(d)]. The weighted FSC^3^ instead recovers the modified point with a small RMS deviation of the spectra for LA fractions down to 0.25. In this analysis, we used *α* = 0 and *β* = 0, except for *f* < 0.5, where *α* = 0.3 was used to obtain convergence. Similar results are obtained using the ‘knock‐out’ FSC^3^ as shown in the Supporting Information.

In this example, we have used the prior knowledge that only two chemical components were present. However, in general, the number of components needed to describe the data is not known a priori, such that an unsupervised analysis needs to determine also the number of components. Generally, we are interested in finding the smallest number of components reconstructing the measured data with an error dominated by random noise. Similar to the results in SVD reported earlier,^1^ the relative reduction of the error when adding an additional component is decreasing with increasing number of components *k*. As a quantitative measure of this relative change, we use the change of the spectral error in units of its standard deviation, yielding the condition

(2) $$||E_{S}^{\left(k\right)}||-||E_{S}^{\left(k+1\right)}||>\eta \sigma \left(E_{S}^{\left(k\right)}\right)\sqrt{P}$$

where *σ*(.) denotes the standard deviation of the argument and *P* is the number of spatial points of the data. The parameter *η* > 0 quantifies the minimum required improvement of the spectral error by an additional component in units of the standard deviation of the spectral error. We use here $\sigma \left(E_{S}^{\left(k\right)}\right)$ instead of $\left||E_{S}^{\left(k\right)}|\right|$ because the weighted algorithm uses the components to reduce not only the global error but also the local error. The combination of both is captured well in the standard deviation.

To find a suited starting value for the number of components, we use the unweighted FSC^3^ with a number of components increasing from *k* = 1 until Eqn (2) is not satisfied. The resulting *k* is then used as starting value for a weighted FSC^3^ iteration, and using the resulting weights an FSC^3^ is performed with an additional component. In case Eqn (2) is fulfilled, the additional component is accepted and we return to the weighted FSC^3^ iteration. Otherwise, one component is removed, and an FSC^3^ using the previous weights is performed, and if Eqn (2) with *k* + 1 replaced by *k* − *1* and $\sigma \left(E_{S}^{\left(k\right)}\right)$ replaced with $-\sigma \left(E_{S}^{\left(k\right)}\right)$ is satisfied, the removal of the component is accepted, and the algorithm goes back to the weighted FSC^3^ iteration. Otherwise, the number of components is taken as final result. A flow diagram of the algorithm is shown in the Supporting Information.

**Figure 2:**
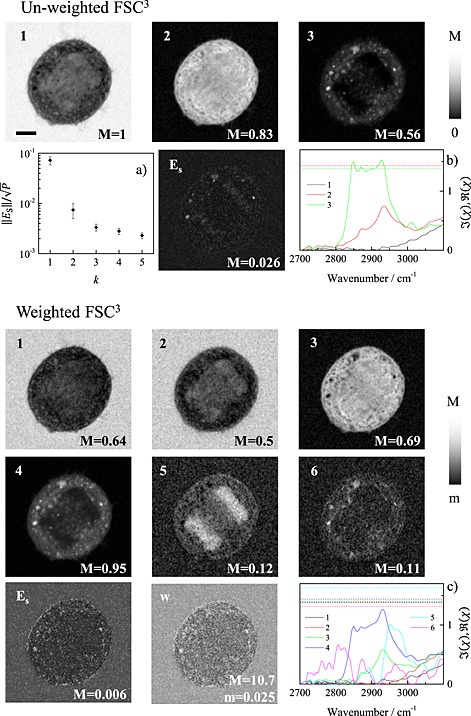
Comparison of the results of the unweighted and weighted FSC^3^ methods using the unsupervised determination of the number of components applied to a CARS hyperspectral image of a fixed U2OS cell. We used *n* = 10, *τ*_H_ = 0.1, and *τ*_L_ = 0.01. Top: unweighted FSC^3^. (a) Spectral error *versus* number of components, the error bars show $\pm \;\eta \sigma \left(E_{S}^{\left(k\right)}\right)$ with *η* = 0.5. The images show the concentration of the components (1–3) and the spectral error **E**_S_ on a linear greyscale as indicated with *m* = 0. Scale bar 5 µm. The component spectra are shown in (b). The solid (dashed) lines are the imaginary (spectrally averaged real) part of the CARS susceptibility. Bottom: weighted FSC^3^ using *α* = 0.3, *β* = 0, *γ* = 10, and *η* = 0.5. Concentration of components 1 to 6, and **E**_S_, with spectra shown in (c). The final weight *w* is shown on a logarithmic greyscale.

We exemplify this algorithm in Fig. 2 for a CARS hyperspectral image of a fixed unstained human bone osteosarcoma epithelial cell (U2OS cell line) undergoing division acquired over the 2600–3700 cm^−1^ range. The medium surrounding the cells was a phosphate‐buffered saline solution. The analysis is applied to the 2700–3100 cm^−1^ region of the retrieved susceptibility, using *η* = 0.5. The initial number of components is determined with the unweighted FSC^3^. The resulting $||E_{S}^{\left(k\right)}||/\sqrt{P}$ and $\eta \sigma \left(E_{S}^{\left(k\right)}\right)$ for increasing *k* are shown in Fig. 2(a). Equation (2) yields *k* = 3. The corresponding concentrations are given in the top part of Fig. 2, showing water (component 1), protein/nucleic acid (component 2), and lipid/protein (component 3). This assignment is consistent with the component spectra given in Fig. 2(b). The spectral error map shows that some smaller spatial structures are not well reproduced by the factorization. The subsequent weighted FSC^3^ iteration results in *k* = 6 components, which are shown in the bottom of Fig. 2. The spectral error is more uniform and has a four times reduced maximum. The first and second components have a similar spatial distribution and spectrum and can be attributed to water. The protein in the cytosol (component 3) is now distinguished from the protein/nucleic acid complex (chromatin) in component 5, which were both parts of component 2 of the unweighted result. This assignment is consistent with the spectra [Fig. 2(c)] with the chromatin shifted to higher wavenumbers compared with the cytosol protein.^16^ Component 6 has a distribution that resembles component 4 (lipid) and a spectral feature over the 2750–2850 cm^–1^ range. It has a rather small maximum concentration of 11%, and we can tentatively assign this component to lipids of higher saturation.^17^ We emphasize that the unweighted FSC^3^ with five components does not separate between the cytosol protein and chromatin complex, and was not converging for more components. More details on the comparison of unweighted and weighted FSC^3^ methods are shown in the Supporting Information.

The FSC^3^ algorithm is also suited to analyze hyperspectral images obtained from other techniques, such as SRS, Raman, and fluorescence. An example of the application of FSC^3^ to hyperspectral SRS of *Caenorhabditis elegans* as well as to hyperspectral confocal Raman images of 3t3l1‐derived adipocytes is shown in the Supporting Information.

### Sparse sampling

In the work of Masia *et al*., 2 we demonstrated a method based on sparse sampling to increase the acquisition speed in sequential hyperspectral imaging while retaining the relevant spectral information. The method first determines the important spectral components of the sample under investigation from a hyperspectral image **d** acquired over a large number of spectral points *S* (sampled at the instrument spectral resolution) but a small number of spatial points using SVD. Subsequently, a limited set of *S*′ spectral positions is determined to retrieve the weights of the important spectral components with the minimum error using a random walk approach. A hyperspectral image **D** is then measured at this small set of spectral positions over a large number of spatial points, and the reconstruction algorithm is applied to generate at each point the large number of spectral points of *S*. For CARS intensity data with a noise scaling as the square root of the intensity, **D** and **d** are taken in this reconstruction as the square root of the measured intensity, which has constant noise.

An important quantity in the reconstruction is the number of components $S_{\text{max}}^{\prime }$ to be used. In the work of Masia *et al.*, 2 $S_{\text{max}}^{\prime }$ was determined considering the increase in the noise by the reduction of spectral points measured and the reduction of the noise by the specific choice of the spectral positions. We found that in some situations, e.g. when the image contains a small number of spatial positions with a particular spectral signature, increasing $S_{\text{max}}^{\prime }$ was improving the reconstruction. We therefore developed a modified method to determine the components used for reconstruction. We still consider the spectral basis given by the left singular spectra **u** of the SVD of **d**, and construct the rotation matrix **û** from the first *S*′ singular spectra of **u** at the selected *S*′ spectral points followed by Gram–Schmidt orthogonalization in sequence of decreasing singular value. Its inverse **û** ^− 1^ is used to reconstruct the data according to **ũû** ^− 1^ **D**, where **ũ** is the (*S* × *S*′) sub‐matrix of **u** containing the first *S*′ singular spectra. For each component, the norm of the corresponding vector in **û** ^− 1^ is the factor by which random noise in **D** translates into the reconstructed data. The modified method limits the amplification of noise by the reconstruction, discarding components for which this norm is more than a factor *ξ* > 1 above the norm of the most significant first component by setting their vector in **û** ^− 1^ to zero, yielding $\tilde{{\hat{u}}^{-1}}$. The resulting reconstructed data with the full spectral range and resolution are then given by $D^{rec}=\tilde{u}\tilde{{\hat{u}}^{-1}}D$.

**Figure 3:**
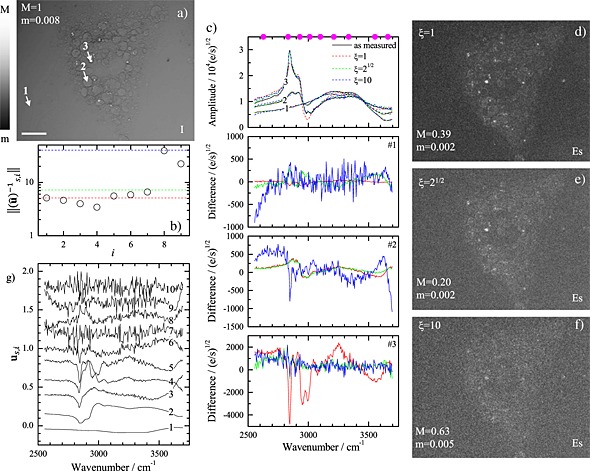
Results of the spectral sampling method in reconstructing the full spectral information from a CARS hyperspectral image of a fixed differentiated embryonic mouse stem cell acquired at nine optimized wavenumbers. (a) Normalized maximum intensity projection of CARS hyperspectral data of a differentiated mouse stem cell in the wavenumber range of 2550–3700 cm^−1^ on a logarithmic greyscale. The scale bar indicates 20 µm. (b) Norm of the vectors of **û** ^− 1^ versus their index. The dashed lines indicate the cutoff for *ξ* = 1 (red), $\xi =\sqrt{2}$ (green), and *ξ* = 10 (blue). (c) Top: measured (solid lines) and reconstructed (dashed lines) square roots of CARS intensities at the positions indicated in (a) for *ξ* as labeled. Bottom: deviation of the reconstructed data at different positions as labeled. (d–f) Spectral error maps for different values of *ξ*, on a logarithmic greyscale as given. (g) First ten singular spectra of the data, vertically displaced for clarity.

An example of the method is given in Fig. 3. The analyzed data are taken on differentiated mouse stem cells imaged using CARS hyperspectral microscopy in the wavenumber range of 2550–3700 cm^−1^ using 5 cm^−1^ steps. A maximum intensity projection of the hyperspectral image is shown in Fig. 3(a). As dataset **D**, we use 1% of the spatial points of the measurement. From the SVD of the square root of the CARS intensity data, we find nine singular value components above the noise 1 and the corresponding spectra in Fig. 3(g). We accordingly used *S*′ = 9 and determined using the random walk approach the set of sparse sampling spectral points **s** [dots in Fig. 3(c)]. The resulting norm of the vectors of **û** ^− 1^ is shown in Fig. 3(b), together with the cutoff level for different values of *ξ*. We reconstruct **D** using the original dataset at nine spectral positions and compare the reconstructed **D** ^rec^ for the three different values of *ξ* with the measured intensity at three different positions indicated by the arrows in Fig. 3(a). The measured (solid lines) and the reconstructed (dashed lines) data are given in Fig. 3(c), together with the residuals. For *ξ* = 1, which leads typically to similar results as the original method^2^ a significant deviation is found at position 3, a small lipid droplet, while for *ξ* = 10, a significant increase of noise is observed. For $\xi =\sqrt{2}$, only the two components with significantly larger norm in **û** ^− 1^ are rejected, compromising between noise and systematic deviation. The spatial distribution of the deviation is visualized in Fig. 3(d)–3(f) by the spectral error $E_{Sp}=\sqrt{P\sum_{j=1}^{S}\;(D_{j,p}-D_{j,p}^{rec}){}^{2}}/∥D{∥}_{F}$, where ∥. ∥ _F_ indicates the Frobenius norm and *P* the number of spatial points in the image. Also, here, $\xi =\sqrt{2}$ produces the smallest spectral error, while for *ξ* = 1, a large spectral error at the positions corresponding to small lipid droplets is found, and for *ξ* = 10, a generally larger spectral error is obtained, as expected from the large norm of the additional components.

### SVD‐based masking

**Figure 4:**
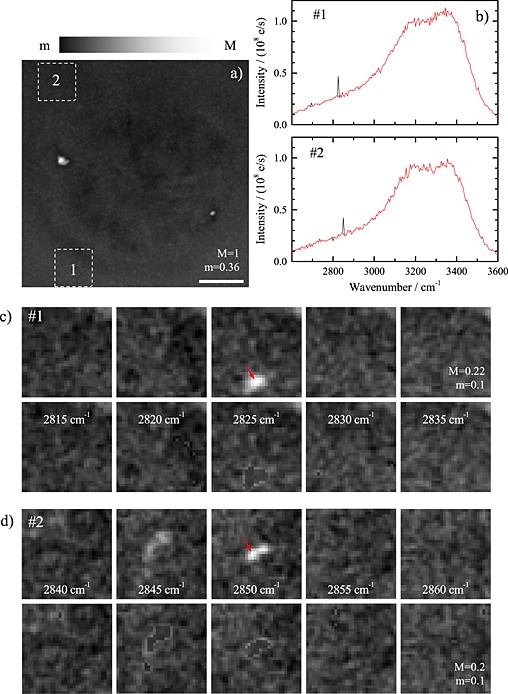
Results of the SVD‐based masking/reconstruction method to filter outliers and motion artifacts in a CARS hyperspectral image of a fixed U2OS cell. (a) Normalized maximum intensity projection of CARS hyperspectral data of a U2OS cell in the wavenumber range of 2600–3600 cm^−1^. The scale bar indicates 5 µm. (b) CARS spectra as measured (black lines) and after reconstruction with *ξ* = 3 and *f* = 0.01 (red lines) corresponding to the spatial position indicated by the red arrows in (c) and (d). (c–d) Measured (top rows) and reconstructed (bottom rows) CARS images of sequential spectral points in the area marked in (a).

Objects in the sample, which are moving during sequential acquisition of hyperspectral data, create motion artifacts in the data. As an example, we show in Fig. 4 hyperspectral CARS data on U2OS cells infected by bacteria. The corresponding maximum projection of the CARS intensity in Fig. 4(a) shows a cross section through a rounded cell close to mitosis, with two lipid droplets. A sequence of five images for consecutive spectral points in the adjacent water regions [see top rows of Fig. 4(c)–4(d)] shows bacteria passing, resulting in spikes in the spectra [black lines in Fig. 4(b)].

Such artifacts in the spectral data are affecting the hyperspectral analysis, and we have developed an iterative algorithm based on SVD to find the corresponding spatial regions and to reconstruct the spectra. We use the noise filtering by SVD^1^ to reconstruct the data using only the spectral components above the random noise. The residual should then be dominated by random noise. Additional fluctuations, like the aforementioned moving objects, can then be identified as points having a residual significantly above the average. In detail, we apply the SVD filtering to the square root of the CARS intensity **D**_*i*_, where *i* is the iteration count, and calculate the residuals $D_{0}-D_{i}^{*}$, where **D**_0_ is the measured square root of CARS intensity and $D_{i}^{*}$ is the SVD‐filtered **D**_*i*_. A data point in **D**_*i* + 1_ takes the value of **D**_0_ when its absolute residual is smaller than $\xi ||D_{0}-D_{i}^{*}||{}_{F}/\sqrt{S\;P}$, with the number of spectral points *S* and spatial points *P*, and a parameter *ξ* > 0. Otherwise, **D**_*i* + 1_ takes the value of $D_{i}^{*}$. The algorithm stops if after an iteration the number of changed excluded points is less than a fraction *f* of the number of excluded points. A workflow of the algorithm is shown in the Supporting Information. An example of the resulting data $D_{i}^{*}$ is shown in the bottom rows of Fig. 4(c)–4(d), showing the removal of the perturbations by the bacteria. This is confirmed by the spectra shown in Fig. 4(b) (red lines), where the spikes have been removed without altering the remaining spectral profile. For the data of Fig. 4, convergence is reached after three iterations using *ξ* = 3 and *f* = 0.01.

## Conclusions

We discussed a number of enhancements to the FSC^3^ analysis of hyperspectral images originally developed for CARS data by Masia *et al.* 1 These are the following: (1) a factorization algorithm, which improves the reconstruction of localized chemical components of small overall concentration by a spatial weighting and determines the number of significant components; (2) a method to determine the number of components for the recovery of the relevant spectral information for sparse sampled data; and (3) a method to filter motion artifacts, which affect the analysis of sequentially acquired hyperspectral images. We demonstrated that (1) allows to reconstruct the presence of a single pixel made of a mixture of 25% linolenic and 75% octanoic acids among 2749 pixels of 100% octanoic acid. We found that in the analysis of data taken on U2OS cells, the new algorithm reconstructed the previously unresolved components of cytosol protein and chromatin complex. We demonstrate that (2) performs better specifically in the situation where a chemical component has a small statistical relevance in a CARS hyperspectral image. The method (3) is based on reconstructing the experimental data, which deviate from the SVD‐filtered data by the SVD‐filtered data.

We emphasize that the FSC^3^ analysis is also applicable to other image contrast techniques beyond CARS, such as SRS, Raman, and fluorescence. In addition to CARS, we show in this work as an example the analysis of an SRS dataset and of a Raman dataset, and we have verified that the method is also suited for fluorescence images. The hyperspectral image analysis software described in this work is available as executable in the Supporting Information. The data presented in this work are available from the Cardiff University data archive under http://dx.doi.org/10.17035/d.2015.100098.

## Supporting information

Supporting info item

Supporting info item
