# Supplementary material for: Hyperspectral image analysis for CARS, SRS, and Raman data
Source: J Raman Spectrosc. 2015 Jun 14;46(8):727–34. doi: 10.1002/jrs.4729 (PMC4950149; doi:10.1002/jrs.4729)
Supplement: Supplementary file 1 — Supporting info item [file JRS-46-727-s001.pdf]

# Hyperspectral image analysis for CARS, SRS, and Raman Supplementary Information

Francesco Masia,* Arnica Karuna, Paola Borri,† and Wolfgang Langbein

*School of Physics and Astronomy, Cardiff University,*

*The Parade, Cardiff CF24 3AA, UK*

*Electronic address: masiaf@cf.ac.uk

†School of Biosciences, Cardiff University, Museum Avenue, Cardiff CF10 3AX, UK

## A. FSC$^3$ reproducibility

The FSC$^3$ algorithm is based on NMF factorization where random initial guesses for concentration and spectra are used. We observed that the FSC$^3$ results are well reproducible in case of simple datasets [1], while for data with more complexity we found a larger distribution of the results. In order to improve the reproducibility we have introduced a modification in the FSC$^3$ algorithm which consists in $n$ NMFs using independent random initial spectra and concentrations, with a high tolerance target $\tau_{\mathrm{H}}$ for fast execution. The NMF with the smallest error is then continued with a low tolerance target $\tau_{\mathrm{L}} \ll \tau_{\mathrm{H}}$. We have tested the improvement in the reproducibility on the datasets of Fig. 2. We have performed 10 FSC$^3$ calculations made either of a single NMF or $n = 20$ NMFs with $\tau_{\mathrm{H}} = 0.1$ and a single NMF with $\tau_{\mathrm{L}} = 0.01$ using the results of the high tolerance calculations as starting point. We quantify the reproducibility using the relative variation $R$ of the reconstructed data $D^*$

$$R = \left\{ \min_i \left[ \operatorname*{rms}_{j \neq i} \left( 2 \frac{||D_i^* - D_j^*||_{\mathrm{F}}}{||D_i^*||_{\mathrm{F}} + ||D_j^*||_{\mathrm{F}}} \right) \right] \right\}, \tag{1}$$

where $i$ and $j$ are the indexes of the FSC$^3$ calculations. Fig. S1 shows $R$ with the two methods for different number of components $K$. The high/low tolerance method (black symbols) gives an improvement in the reproducibility of 3-8 times with respect to the single NMF method (red symbols) depending on the number of components $K$. Alternative to the "high/low tolerance" method, we have developed a "knock-out" method showing further improved reproducibility. In the "knock-out" method, we run a set of $2^n$ NMF calculations with tolerance $\tau$ using random initial guesses. We then select the half of the solutions with the smaller errors and use them as initial conditions for the next set of NMF calculations with same target tolerance $\tau$. We repeat this until we obtain a single solution. In the algorithm there is the option to compare the last two solutions for similarity, for which we calculate the relative error between the solutions

$$\epsilon = \frac{||D_1^* - D_2^*||_{\mathrm{F}}}{||D_1^* - D||_{\mathrm{F}} + ||D_2^* - D||_{\mathrm{F}}}, \tag{2}$$

where $D_{1,2}^*$ are the reconstructed data obtained from the two solutions and $D$ are the original data. If $\epsilon$ is larger than a user defined maximum relative error $\epsilon_{\mathrm{max}}$, a new set of $2^n$ calculations is started and the final two solutions of the iteration is added to the previous final two and out of them the two solutions with the lowest error are retained for the similarity check.

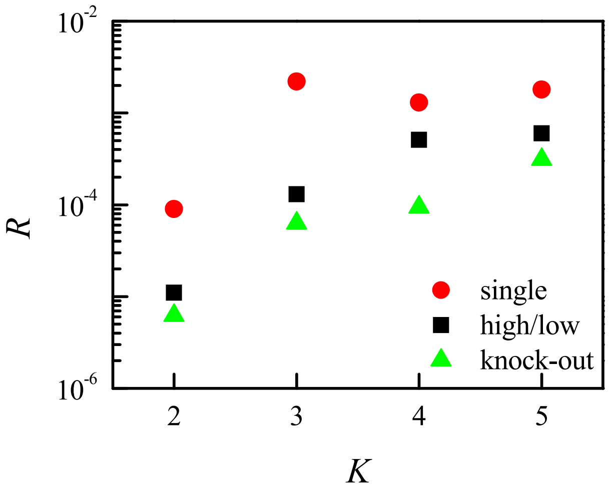


FIG. S1: Comparison in the reproducibility of the results versus number of components $K$. Relative variation $R$ of the reconstructed data considering a single NMF (red), the high/low tolerance (black symbols) and the "knock-out" method (green symbols).

As can be seen in Fig. S1, the "knock-out" method shows an improved reproducibility with respect to the "high/low" method by about a factor of two. For the analysis we used $n = 4$, $\tau = 0.1$ and $\epsilon_{\max}= 0.01$.

### B. FSC$^3$ flowchart

Fig. S2 shows the flowchart of the weighted FSC$^3$ algorithm. The red dashed blocks represent individual FSC$^3$ calculation. $K$ is the number of component used, and the binary vector $e$ defines if a particular spatial pixel has to be excluded ($e = 1$) or included ($e = 0$) in the analysis. The algorithm returns the last spectra and concentrations saved.

### C. Comparison of the un-weighted and weighted FSC$^3$ algorithm using the "high/low tolerance" method

In this section we compare the results of the un-weighted and weighted FSC$^3$ methods applied to the hyperspectral images of Fig. 2 for different numbers of components.

Figures S3-S5 show the results of the un-weighted FSC$^3$ method for $K = 3$, 4 and 5, respectively. In Fig. S3 the hyperspectral data are factorized as water (component 1), protein (component 2) and lipid (component 3). The spectral error is large for the lipid droplets

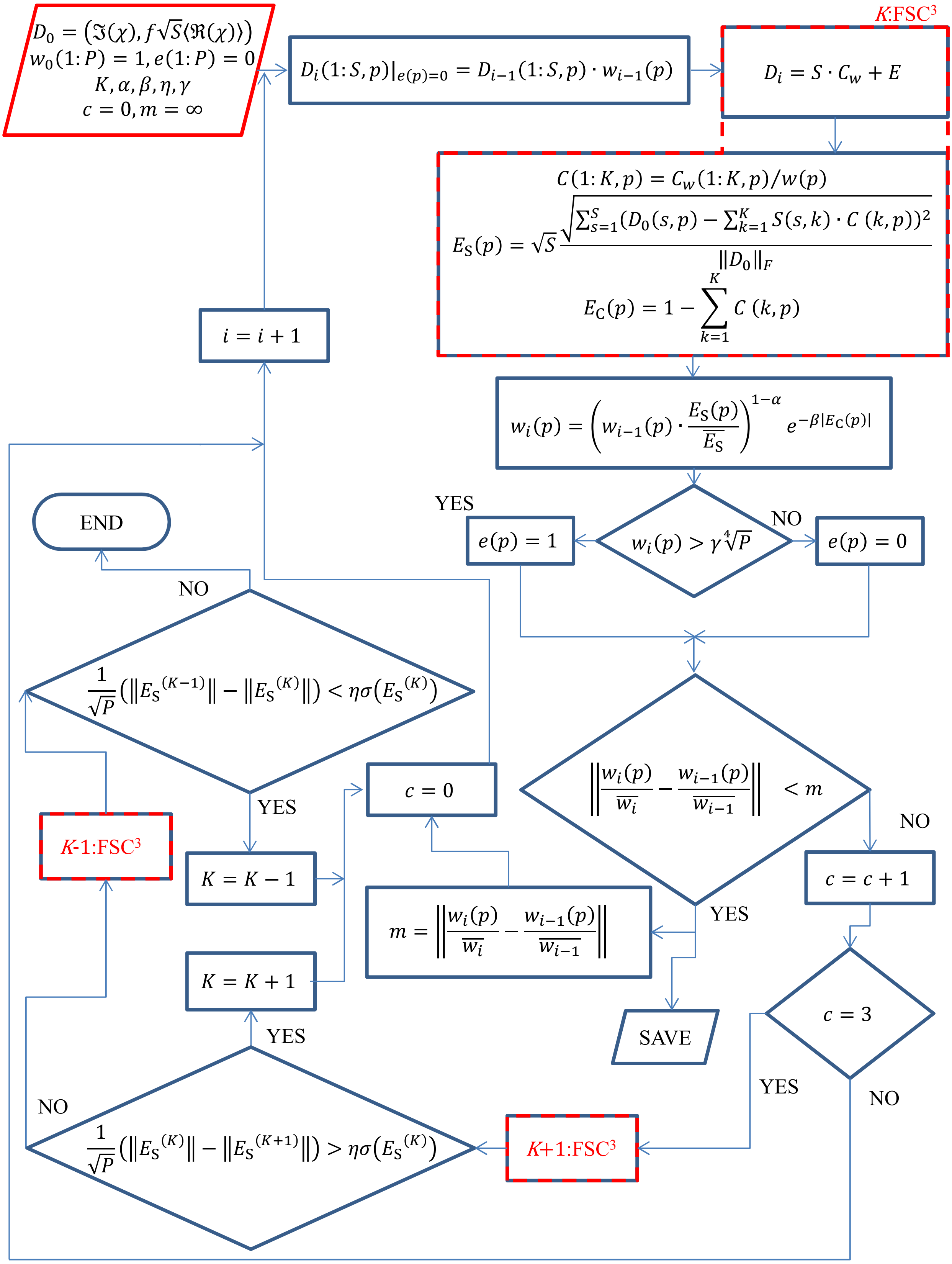


FIG. S2: Flowchart of the weigthed FSC$^3$ algorithm.

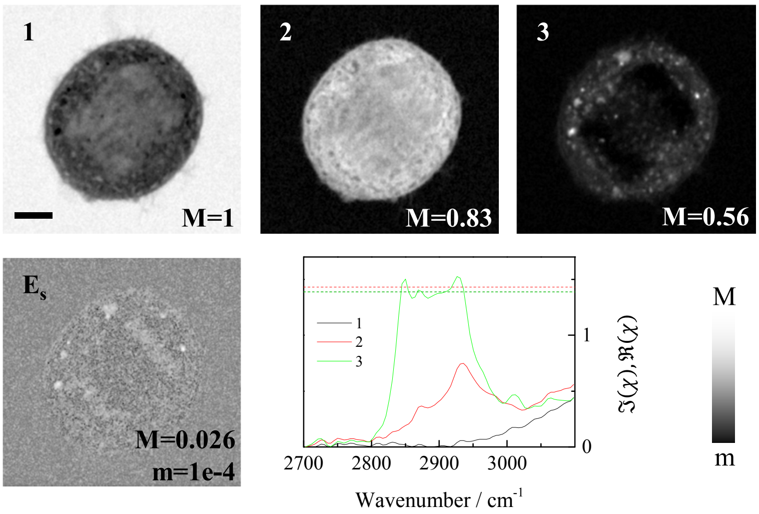


FIG. S3: Results of an un-weighted FSC$^3$ analysis on the hyperspectral data of Fig. 2 using $K = 3$ components. Concentration maps are shown on a linear gray scale with maximal values $M$ as given. Corresponding spectra are shown in the plot. The solid (dashed) lines are the imaginary (real) part. The spectral error $\mathbf{E}_\mathrm{S}$ is shown on a logarithmic gray scale as indicated.

and the chromatin. Increasing the number of components to 4 (see Fig. S4) separates the chromatin (2) to some extend, as suggested from the spectrum which is blueshifted from the cytosol protein (component 4), typically observed in nucleic acids. The extended spatial distribution of component 2 suggests that the standard FSC$^3$ is not able to distinguish completely the chromatin from the cytosolic protein. Adding an additional component (see Fig. S5) results in the water being distributed into two components (1 and 2) with no variation in the other components. The spectral error still shows that the data at the lipid droplet are not well represented.

Figures S6-S9 summarize the results of the weighted FSC$^3$ algorithm for 3 to 6 components, including the final weights $w$. In general the components obtained with the weighted algorithm using only 3 components are similar to the ones of Fig. S3. The component 2 (protein/nucleic acid) in Fig. S6 has a spatial distribution which is more structured than the corresponding component of Fig. S3, with larger concentration in the area of the DNA. The spectral error is more homogeneous than what obtained in the un-weighted method and the weight map shows larger values at the lipid droplets and chromatin regions, indicating that these areas are not well reproduced. The correlation with the spectral error shows that too few components were used.

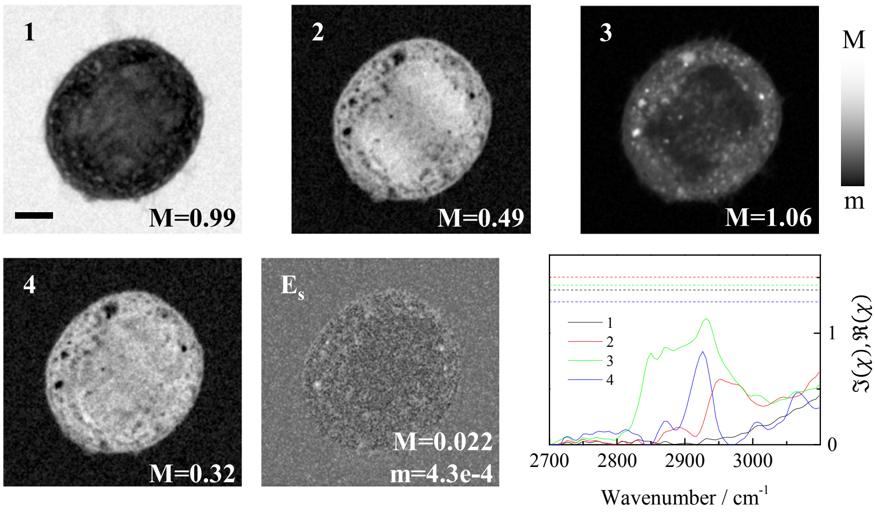


FIG. S4: Same as Fig. S3 with $K = 4$.

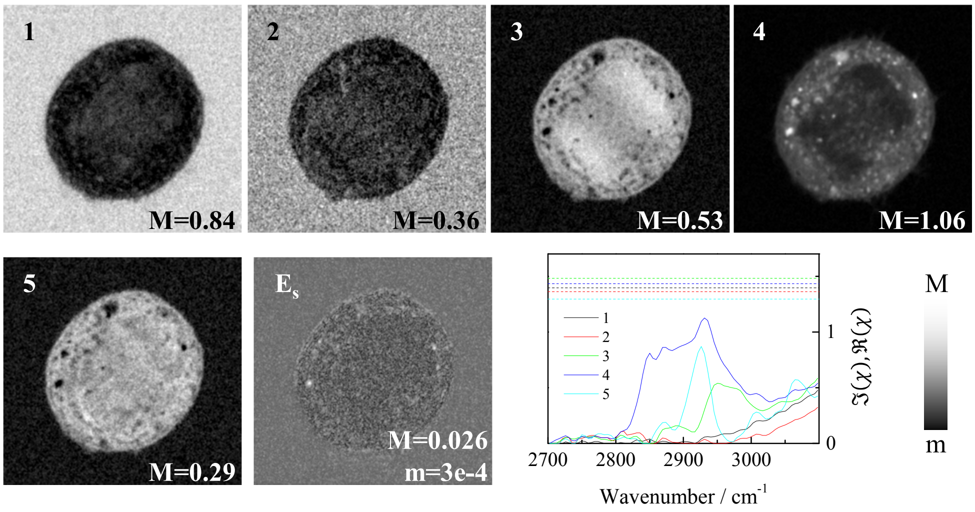


FIG. S5: As Fig. S3 with $K = 5$.

In the next step of the iteration the weighted FSC$^3$ method increases the number of components $K$ to 4 (see Fig. S7), and distinguishes chromatin as an additional component (4). The corresponding spectrum is blueshifted compared to the cytosolic protein (component 2) consistent with nucleic acids.

In the subsequent steps $K$ is further increased to 5 ((Fig. S8) and then 6, (Fig. S9) which is the final result of significant number of components. Component 4 in Fig. S8 is distributed in the water and the cell, but is separated in Fig. S9 into component 6 and the water components 1,2. Component 6 is now present mainly within the cell and presents a spectral feature which extends in the (2750-2850) cm$^{-1}$ range and can be tentatively assigned to

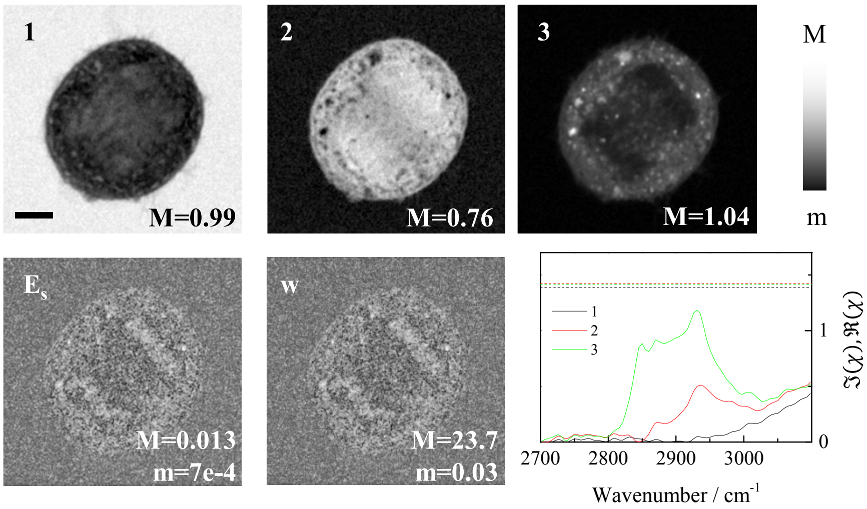


FIG. S6: As Fig. S3, but for a weighted FSC$^3$ analysis. $m = 0$ except for the resulting spectral error $\mathbf{E}_S$ and weight $w$ shown on logarithmic gray scales as indicated.

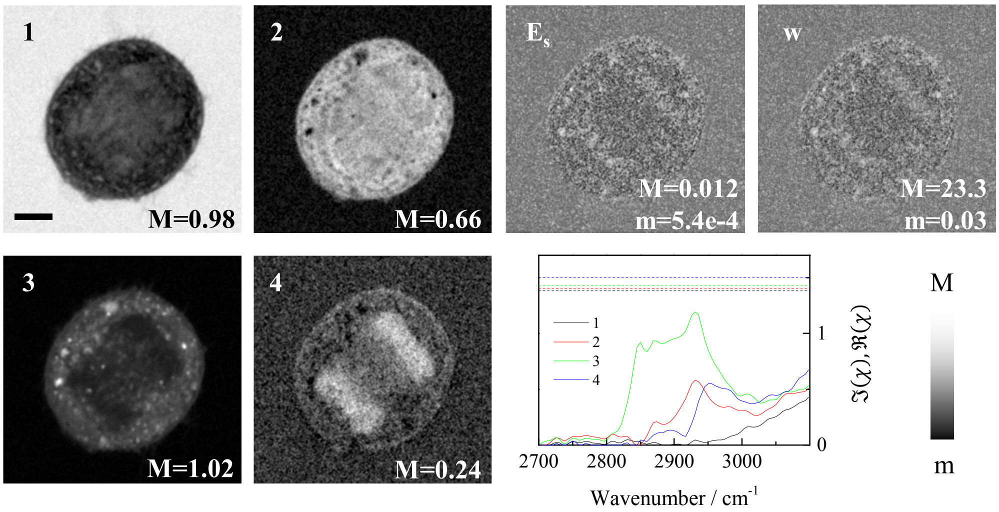


FIG. S7: Same as Fig. S6 with $K = 4$.

lipids with high saturation. The water components 1 and 2 are both present outside the cell in similar concentrations, but separated in the chromatin / spindle region - component 1 in the center of the spindle, and component 2 at the end of the spindles. This suggests that they are different modifications of the water spectrum in the solvation layers around the membranes / proteins.

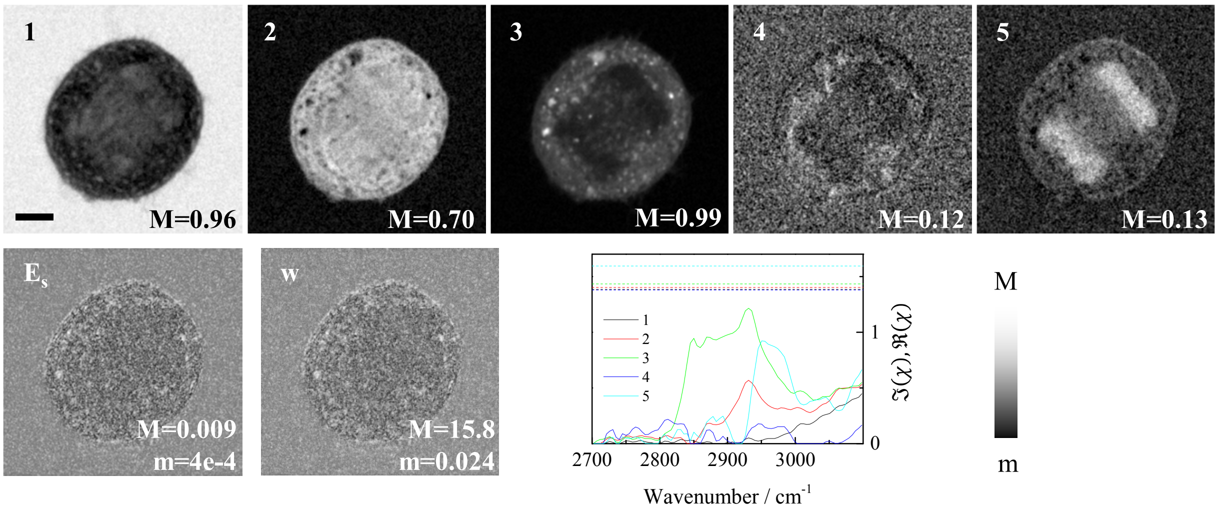


FIG. S8: Same as Fig. S6 with $K = 5$.

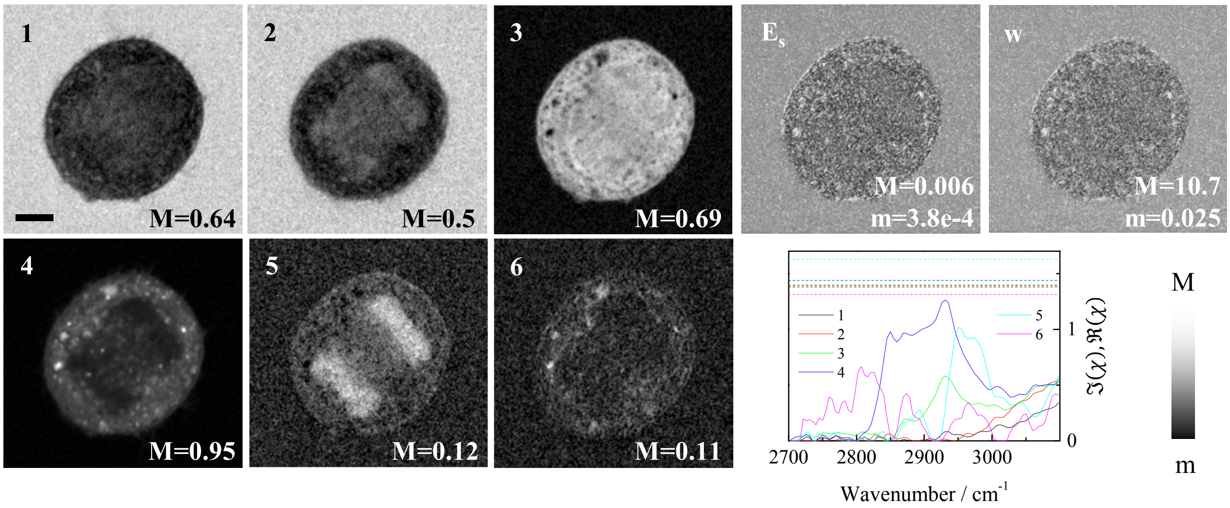


FIG. S9: Same as Fig. S6 with $K = 6$.

### D. Comparison of the un-weighted and weighted FSC$^3$ algorithm using the "knock-out" method

Figure S10 shows the comparison of the un-weighted and weighted FSC$^3$ algorithm using the "knock-out" method on the lipid mixture data of Fig. 1. For the analysis we used $K = 2$, $n = 3$ and tolerance $\tau = 0.1$ and $\tau = 0.001$. No check of the similarity of the last two solutions was performed during the knock-out process. For the weighted algorithm we used same parameters as for the "high/low" tolerance method ($\beta = 0$, $\gamma = 10$, with $\alpha = 0$ except for $f < 0.5$ where $\alpha = 0.3$ was used for convergence). The results are similar to

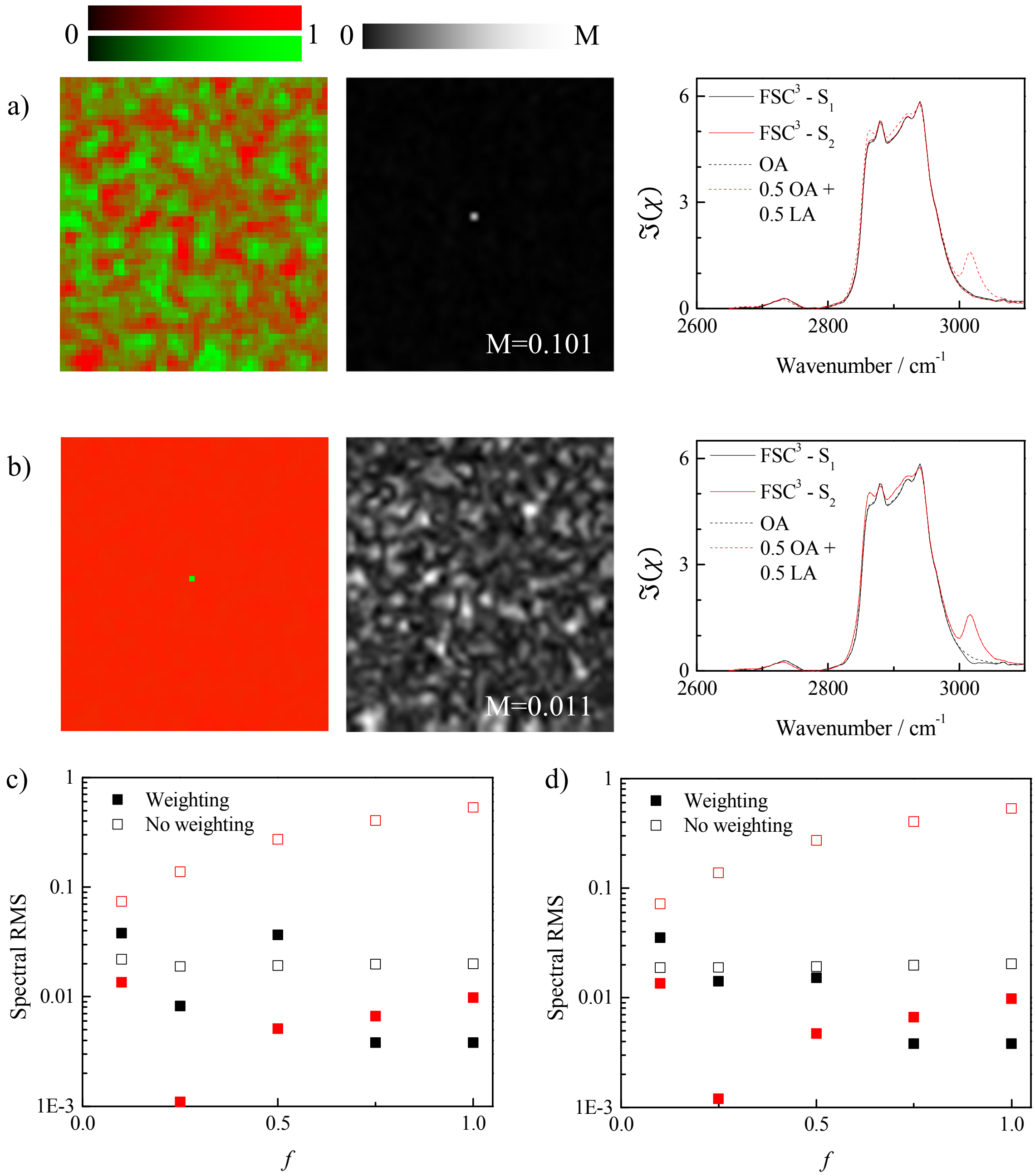


FIG. S10: Comparison of the performance of the un-weighted and weighted FSC$^3$ method in case of a synthetic CARS hyperspectral image made with two lipids using the "knock-out" FSC$^3$ method. a-b) Concentration maps (left) of the two FSC$^3$ components mapped into the red and green value, spectral error (middle) and spectra (right) of the corresponding components (solid lines) and pure substances (dashed lines). Black (red) lines are the first (second) component and OA (LA), respectively. a) standard FSC$^3$ b) weighted FSC$^3$. M indicates the maximum value of the greyscale. c-d) RMS deviation of the FSC$^3$ spectra from to the pure substance spectra for the un-weighted (empty symbols) and weighted FSC$^3$ (full symbols) as a function of the LA fraction $f$ in the modified point. Black (red) symbols refer to the first (second) FSC$^3$ spectrum. c) $\tau = 0.1$ d) $\tau = 0.001$.

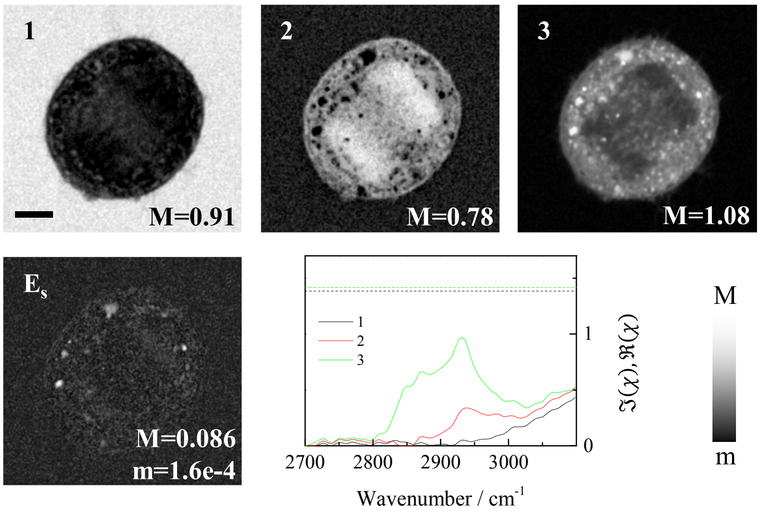


FIG. S11: Results of an un-weighted FSC$^3$ analysis on the hyperspectral data of Fig. 2 using automatic determination of the number of components and the "knock-out" method. Concentration maps are shown on a linear gray scale with maximal values $M$ as given. Corresponding spectra are shown in the plot. The solid (dashed) lines are the imaginary (real) part. The spectral error $\mathbf{E}_\mathrm{S}$ is shown on a logarithmic gray scale as indicated.

the one obtained with the "high/low tolerance" method (see Fig. 1) showing that only the weighted algorithm is able to identify the pixel with modified spectrum for $f$ down to 0.125.

Figs S11 and S12 show a comparison between un-weighted and weighted FSC$^3$ algorithm using the "knock-out" method and automatic determination of the number of chemical components for the data of Fig. 2. In the case of the un-weighted FSC$^3$ (Fig. S11) the algorithm returns $K = 3$ components which can be assigned to water, protein/chromatin and lipid/protein. Similarly to the "high/low tolerance" method (see (Fig. 2) the chromatin is not distinguished from the rest of cytosolic proteins. Using the weighted FSC$^3$ (Fig. S12) the algorithm returns $K = 5$ components which include water, cytosol protein, lipid and chromatin, with a fifth component distributed mostly in the lipid droplets, which can indicate higher saturation degree of the lipids. For the analysis we used $n = 3$, $\tau = 0.1$, $\epsilon_{\mathrm{max}}= 0.01$, $\eta = 0.5$, and for the weighted algorithm $\alpha = 0.3$, $\beta = 0$ and $\gamma = 10$.

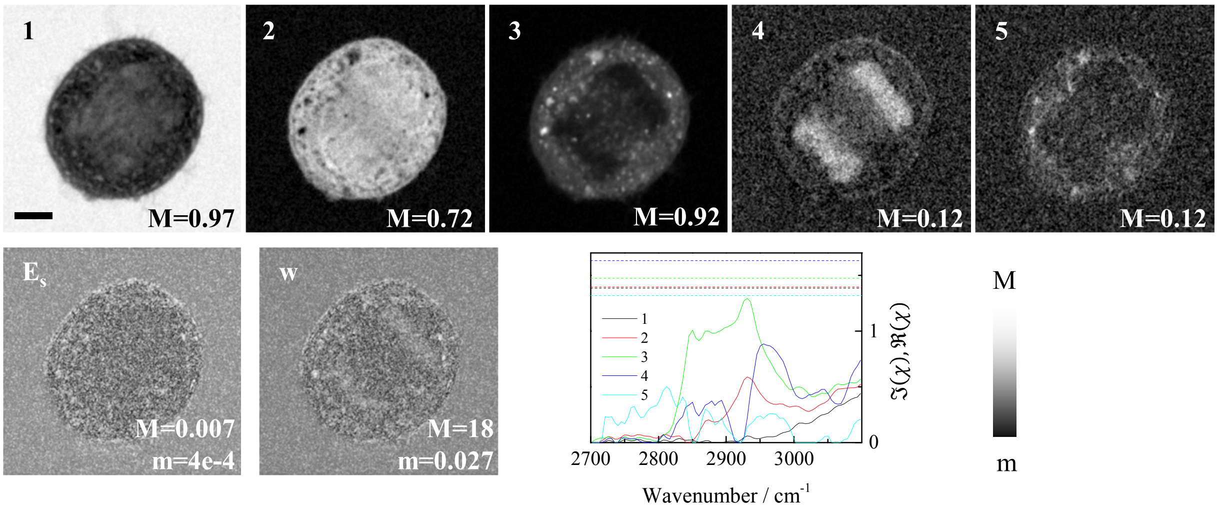


FIG. S12: Same as Fig. S11, but for a weighted FSC$^3$ analysis. $m = 0$ except for the resulting spectral error $\mathbf{E}_S$ and weight $w$ shown on logarithmic gray scales as indicated.

### E. Analysis of SRS hyperspectral images using FSC$^3$

The FSC$^3$ algorithm can be used to analyze hyperspectral images obtained with techniques different from CARS. Here we report the analysis of SRS hyperspectral data of *C. elegans* acquired in the region (1620-1800) cm$^{-1}$.[2] The SRS signal is proportional to the imaginary part of the susceptibility so that we skip the SVD filtering and PCKK retrieval method in the analysis. The FSC$^3$ factorization is applied directly on the measured SRS signal. The concentrations of the single components are individually normalized to a maximum of one and cannot be determined absolutely in the same way as for CARS since SRS does not provide a contrast for substances with no resonances in the investigate wavenumber range, which is mostly water for this data. The component spectra are normalized accordingly to retain the factorized data values. Fig. S13 shows the concentration and spectra obtained using FSC$^3$ with $K = 4$ chemical components. The results are consistent with results on a similar sample obtained with MCR analysis [2]. Component 4 can be associated to unsaturated lipids such a glyceryl trioleate [3] by the presence of the resonances of the acyl C=C bond at 1655 cm$^{-1}$ and the weaker ester C=O bond at 1745 cm$^{-1}$. Following the attribution in Ref. [2], component 3 corresponds to lysosome related organelles, component 1 to protein with the broad amide I band around 1650 cm$^{-1}$, and component 2 to oxidized lipids with a strong band at the ester bond. The spectral error in Fig. S13 is homogeneous and shows

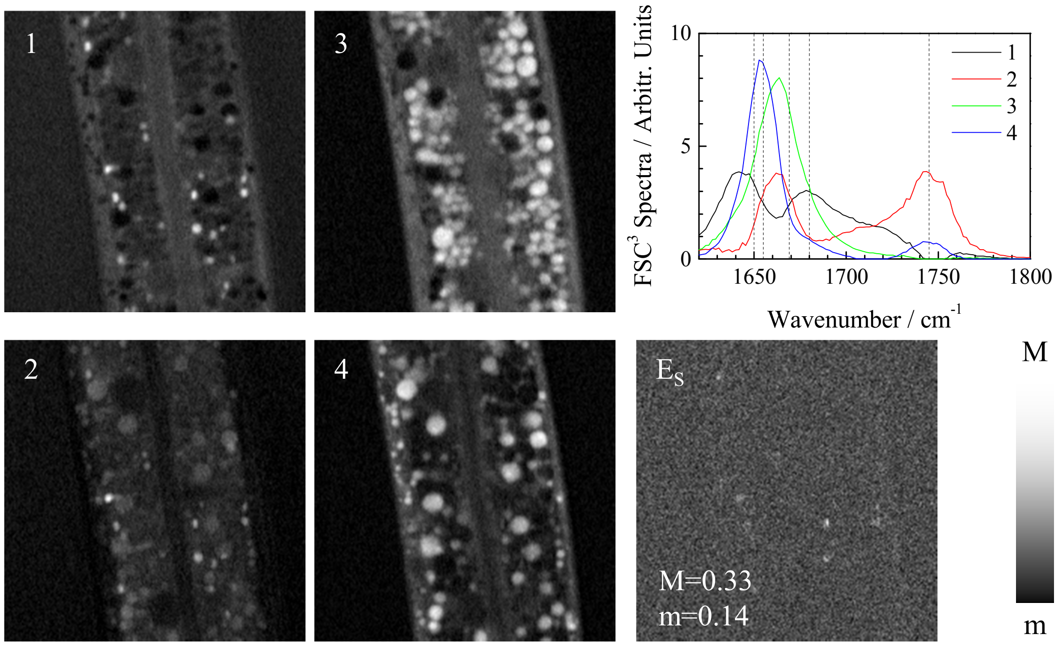


FIG. S13: FSC$^3$ analysis of hyperspectral SRS data of *C. elegans*. The concentration maps (images 1-4) have a linear gray scale with $M = 1$ and $m = 0$. The spectra of the corresponding component are shown in the plot. The vertical dashed lines indicate the wavenumbers 1650 cm$^{-1}$(amide I), 1655 cm$^{-1}$(acyl C=C bond), 1669 cm$^{-1}$ (sterol C=C group of cholesterol), 1680 cm$^{-1}$(aldehyde group) and 1745 cm$^{-1}$(esther C=O bond). The spectral error $\mathbf{E}_\mathrm{S}$ is shown with a gray scale between 0.14 and 0.33.

little spatial structure, demonstrating the ability of the FSC$^3$ method to factorize the data and remove the noise. This factorization took about 20 seconds on a modern desktop PC, much faster than the MCR analysis used in [2].

### F. FSC$^3$ analysis on spontaneous Raman hyperspectral images

3t3l1-derived adipocytes have been imaged with spontaneous Raman scattering imaging in confocal geometry on the same microscope used for CARS measurements [3]. Raman signal has been excited using a continuous-wave 532 nm laser focused by the 20× 0.75 NA dry objective, which has been used also for collection. The laser line is filtered by a combination of a dichroic (Semrock LPD01-532RS-25) and low pass (Semrock BLP01-532R-25) filters. The emitted intensity is dispersed by an imaging spectrometer (Horiba Jobin-Yvon iHR 550) with a a 150 lines/mm grating, and detected by a cooled back-illuminated CCD

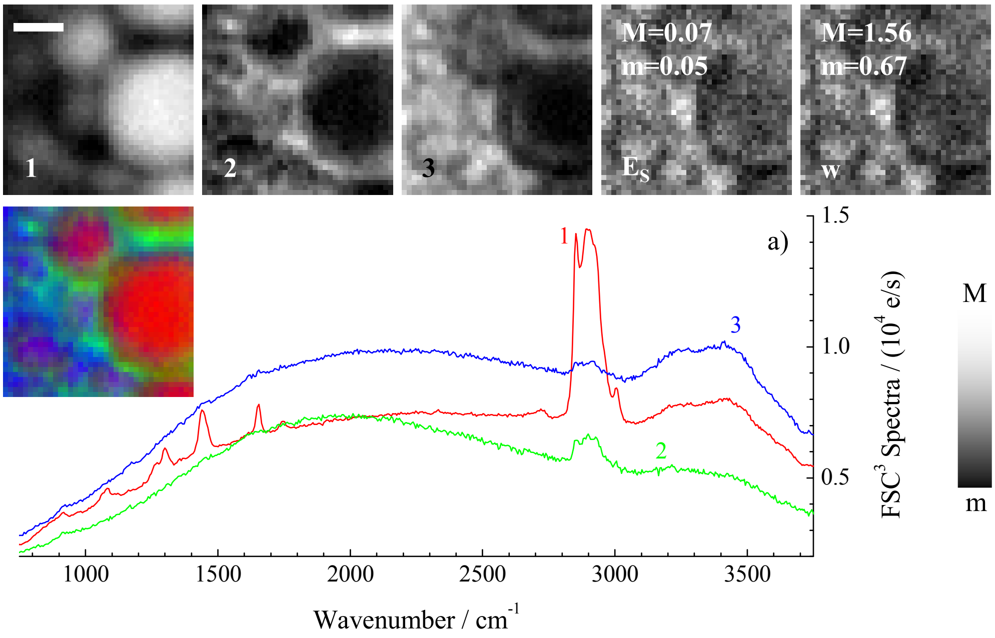


FIG. S14: FSC$^3$ analysis of hyperspectral confocal Raman images of 3t3l1-derived adipocytes. The images contain $38 \times 38$ square pixels of $0.5\,\mu$m size. The scale bare corresponds to $5\,\mu$m. The concentration maps (images 1-3) have a linear gray scale with $M = 1$ and $m = 0$. The spectra of the corresponding component are shown in the graph, as photoelectron rate per channel. The spectral error $\mathbf{E}_\mathrm{S}$ and final weight $w$ distributions are also shown using the indicated ranges. The RGB image has been constructed using the concentration maps of the first, second and third component as red, green and blue channel, respectively.

(Andor Newton DU 971N). The spectra were acquired using a gain of $4\times$ and the 1 MHz digitizer, using a pixel dwell time of 0.2 s. The data have been corrected by the spectral sensitivity of the grating. An un-weighted FSC$^3$ calculation has been performed on the intensity using n=50, $\tau_\mathrm{H} = 0.01$ and $\tau_\mathrm{L} = 0.001$. The number of components is found to be $K = 3$ for $\eta = 0.25$. The results of the weighted algorithm is shown in Fig. S14. The optimal number of components found was also $K = 3$. Similarly to the analysis on SRS data, no constrain on the concentration sum has been used and the obtained concentration distributions have been normalized individually to a maximum of one. The spectra have been corrected correspondingly.

All spectra show a significant fluorescence background. From the concentration image and the spectrum, we can identify component 1 as lipid, similar to GTO. The image shows a larger and some smaller lipid droplets. Component 2 is dominated by fluorescence and is

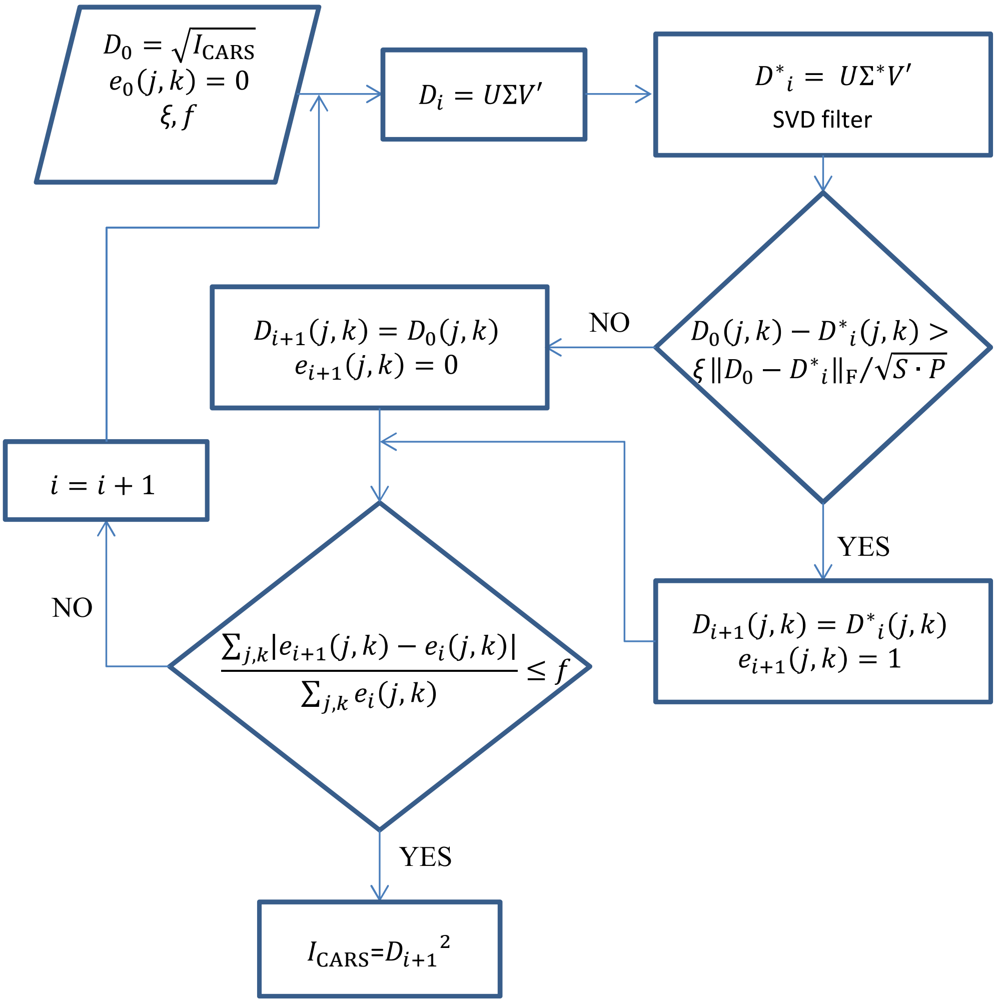


FIG. S15: Flowchart of the SVD based masking algorithm.

localized at the lipid droplet membrane. It contains some water and signal in the CH stretch region (2800-3100) cm$^{-1}$, and weak features in the characteristic region (750-1800) cm$^{-1}$. Component 3 is also dominated by fluorescence and is localized in the cytosol. It contains more water than component 2, and a weaker but spectrally similar feature in the CH-stretch region.

### G. SVD based masking flowchart

In Fig. S15 the flow chart of the SVD based masking algorithm is shown. The binary vector $e$ defines if a particular point has to be excluded from the SVD factorization at the next iteration step.

### H. Running the Hyperspectral Imaging Analysis (HIA) software

The software is attached as supplementary information to the paper. First download the "HIA_Software_JRS.zip.pdf" and rename it as a zip file. Extract all the files in a folder. The software is written in MATLAB, compiled for Windows 7 64bit, and requires the MATLAB run-time compiler version 8.3 (2015a) to be installed. The java class "DropTargetList.class" and the parallel computing profile "HIA.settings" must be in the same folder as the executable. To run the software, launch the batch file "run_HIA.bat". This will open a cmd window which can show any MATLAB error occurred during the analysis. Please use this feature to identify and report bugs. The file "HIA.chm" is a help file with the instructions to use the program and the description of the functions.

---


[1] F. Masia, A. Glen, P. Stephens, P. Borri, and W. Langbein, *Anal. Chem.* **2013**; *85*, 10820.

[2] P. Wang, B. Liu, D. Zhang, M. Y. Belew, H. A. Tissenbaum, and J.-X. Cheng, *Angew. Chem. Int. Ed.* **2014**; *53*, 11787.

[3] C. Di Napoli, F. Masia, I. Pope, C. Otto, W. Langbein, and P. Borri, *J. Biophotonics* **2014**; *7*, 68.
